# Would a Glass of Wine Hurt Me?

**Published:** 1854-02

**Authors:** 


### WOULD A GLASS OF WINE HURT ME?

This is a question often proposed to me as a physician. the patient honestly supposing that it would strengthen him. My uniform reply is,

Substantial, reliable strength is only to be derived from the perfect digestion of plain, nutritious food; all else is transient, fictitious, and worse than useless, as it ultimately, and that within a few hours, always and inevitably makes them weaker than before.

Better a thousand-fold let a man die of ordinary disease than risk making him a drunkard, in restoring him to health by the use of alcohol in any form, even if it could be done.

The use of stimulating drinks, and they are used only because they do stimulate, are wholly pernicious to the young, even when of the purest and best of their kind, but immeasurably more destructive when of the kind referred to in the January number of "*The Prohibitionist,*" published monthly, at fifty cents a year, at Albany, N. Y., under the head of "A DEATH-BED REVELATION."

"A large Wine Dealer residing in London recently, on his death-bed, being in great distress of mind, acknowledged to his friends that his agony was occasioned by the nature of the business he had followed for years. He stated that it had been his habit to purchase all the sour wines he could, and by making use of sugar of lead, and other deleterious substances, restore the wine to a palatable taste. He said he did not doubt he had been the means of destroying hundreds of lives, as he had from time to time noticed the injurious effects of his mixtures on those who drank them. He had seen instances of this kind where the unconscious victims of his cupidity, after wasting and declining for years, despite the best medical advice, went to their graves, *poisoned by the Adulterated Wines* he had sold them.

This man died rich, but alas, what legacy did he leave for his children! Wealth gotten by deceit, and that not of a harmless, but fatal nature."

From an extended and varied observation of twenty-five years, I have arrived at the stereotype advice to all who consult me as to my opinion of the value of the daily use of a small amount of pure wines, or cordials, or brandies, that—

ANY MAN WHO ONCE drinks a drop of "liquor," MAY DIE IN THE GUTTER; he who tastes it NEVER, NEVER CAN.

